# Video-assisted thoracic surgery for superior posterior mediastinal neurogenic tumour in the supine position

**DOI:** 10.4103/0972-9941.55110

**Published:** 2009

**Authors:** Laleng M Darlong

**Affiliations:** Department of Surgery, North East Indira Gandhi Regional Institute of Health & Medical Sciences, Shillong, India

**Keywords:** Superior posterior mediastinum, supine position, video-assisted thoracic surgery

## Abstract

Video-assisted thoracic surgery (VATS) for a superior posterior mediastinal lesion is routinely done in the lateral decubitus position similar to a standard thoracotomy using a double-lumen endotracheal tube for one-lung ventilation. This is an area above the level of the pericardium, with the superior thoracic opening as its superior limit and its inferior limit at the plane from the sternal angle to the level of intervertebral disc of thoracic 4 to 5 vertebra lying behind the great vessels. The lateral decubitus position has disadvantages of the double-lumen endotracheal tube getting malpositioned during repositioning from supine position to the lateral decubitus position, shoulder injuries due to the prolonged abnormal fixed posture and rarer injuries of the lower limb. There is no literature related to VATS in the supine position for treating lesions in the posterior mediastinum because the lung tissue falls in the dependent posterior mediastinum and obscures the field of surgery; however, VATS in the supine position is routinely done for lesions in the anterior mediastinum and single-stage bilateral spontaneous pneumothorax. Thus, in the selected cases, ‘VATS in supine position’ allows an invasive procedure to be completed in the most stable anatomical posture.

## STANDARD

The superior posterior mediastinum is an area above the level of the pericardium, with the superior thoracic opening, its superior and inferior limits at the plane from the sternal angle to the intervertebral disc of thoracic 4 to 5 vertebra lying behind the great vessels. VATS for a superior posterior mediastinal lesion is routinely done in the lateral decubitus position similar to a standard thoracotomy using double-lumen endotracheal tube for one-lung ventilation. The lateral decubitus positioning of the patients has disadvantages of the tube getting displaced during repositioning of the patient from the supine position to the lateral decubitus position, shoulder dysfunction and injuries of the lower limb.[1] The supine positioning of the patients for a lesion in the superior posterior mediastinum thus reduces some of the disadvantages associated with a lateral decubitus positioning. There is no literature available that describes ‘VATS in the supine position’ for lesions in the posterior mediastinum because the lung tissue falls in the dependent posterior mediastinum in the supine position and obscures the field of surgery; however, VATS in the supine position is otherwise routinely done for lesions in the anterior mediastinum and for single-stage bilateral spontaneous pneumothorax.[2]

## OUR MODIFICATION

**Figure 1:**
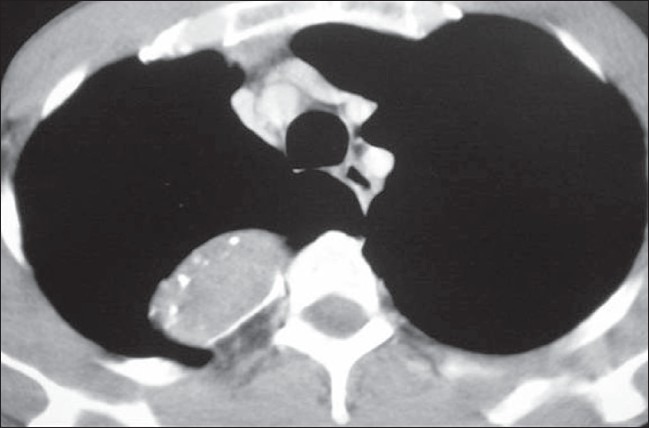
CT scan of the thorax showing a posterior mediastinal neurogenic tumour with no intraspinal extension

In our modification for approaching a superior posterior mediastinal neurogenic tumour on the right side of the thorax, instead of the routine lateral decubitus position, we positioned the patient supine. Our idea was to take advantage of the apical location of the neurogenic tumour and to extract its specimen using a mini-axillary thoracotomy wound. In this way, we also avoided complications arising due to a change in posture on the double-lumen endotracheal tube for one-lung ventilation and shoulder dysfunction described with the lateral decubitus position. The neurogenic tumour was located on the right side of the thorax extending from the apex of the thoracic cavity to the base lying over the second and third costovertebral junction with a dimension of 7 × 5 cm with no intraspinal extension [Figure 1].

**Figure 2:**
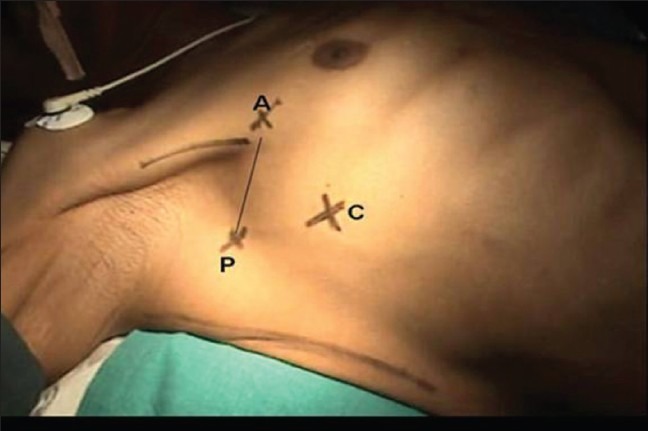
Patient in the supine position with the limb abducted and port sites; A, anterior axillary line in the third intercostals space; P, posterior axillary line along the fourth intercostals space; C, camera port in the fifth intercostals space along the mid-axillary line, point A and P are joined together for a utility mini-axillary thoracotomy

**Figure 3:**
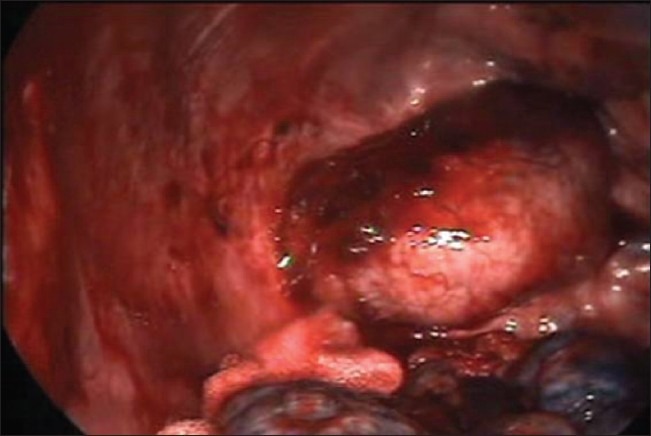
The superior posterior mediastinal neurogenic tumor with the lung displaced inferiorly

**Figure 4:**
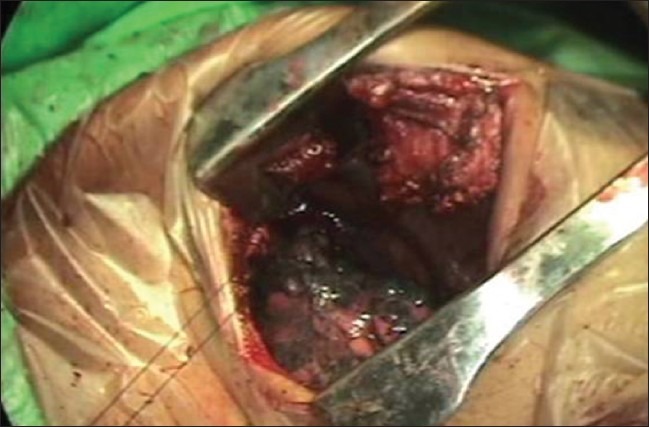
Utility mini-axillary thoracotomy wound used for dissection and specimen extraction

One-lung ventilation using a double-lumen endotracheal tube was used for the isolation of the lung. With the patient already in the supine position, we achieved further widening of the rib space in the anterior and lateral chest wall by placing sandbags in-between the shoulder blades. The upper limb on the side of the lesion is abducted to 90 degrees thus exposing the axilla well. Following this, the anterior and posterior axillary folds become well marked and prominent. The lung on the side of the lesion is collapsed thus allowing easy access and improved visualization of the superior posterior mediastinum. The camera port is sited at about the fifth intercostal space in the mid-axillary line using a 1.5 cm incision. Two additional working ports of size 1.5 cm are also made, one at the third intercostal space along the anterior axillary line and another at the fourth intercostal space along the posterior axillary line [Figure 2]. Using these two working ports, adhesionolysis and further decompression of the lung tissue are done, so as to isolate the lung inferiorly and free the superior mediastinal mass. For the further displacement of the lung inferiorly, the patient can be positioned in a slight reverse Trendelenburg position thus taking advantage of its superior posterior mediastinal location [Figure 3]. Following this, an attempt to dissect smaller lesion with a narrow base from the posterior mediastinum can be done. However, for a lesion with a broad base or size > 6 cm, an 8 cm utility mini-axillary thoracotomy [Figure 4] is done by joining the two port sites in the anterior and posterior axillary lines [Figure 2]. In our case, because of the size of the lesion, this mini-axillary thoracotomy was made following removal of lung adhesion from the tumor. This utility mini-axillary thoracotomy site is then used as a working channel which normally would have been required even if dissection was completed via thoracoscope for the extraction of the specimen.[3,4] The other advantage of this utility mini-axillary thoracotomy allowed the use of normal conventional, long, hand instruments without restriction when using through the 1.5 cm port wound. Following the complete extraction of the specimen through the mini-axillary thoracotomy wound and haemostasis, a chest tube was inserted through a stab wound below the camera port. The mini-axillary thoracotomy wound which is approximately 8 cm in length is then closed. Thus with the supine positioning of the patient we were able to complete an invasive procedure in the most anatomically stable position.

## BENEFITS

The benefits of a supine positioning for a superior posterior mediastinal tumour are the result of avoiding the lateral decubitus position and taking advantage of the apical location of the lesion.

**Figure 5:**
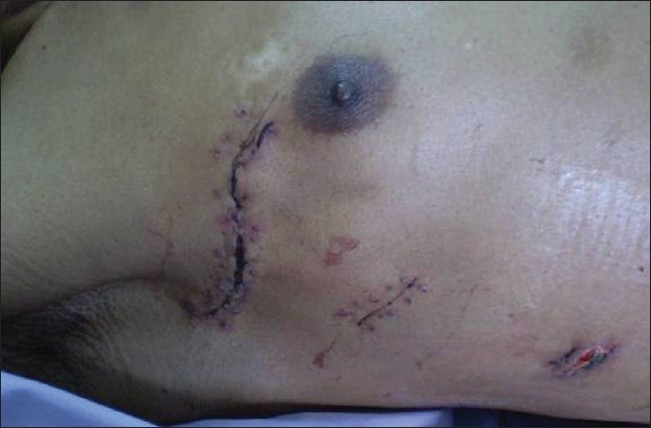
Postoperative showing wound in the axilla

The VATS in the supine position minimizes the risk of displacement of the double-lumen endotracheal tube which can occur during positioning of the patient in the lateral decubitus position which can be substantial and even life threatening.[1] Supine positioning of the patient also avoids the risk of compression injuries on the shoulder during the lateral decubitus position like injury to the axillary nerve due to the weight and fixed prolong awkward posture.[5,6] The less encountered sciatic nerve palsy and myonecrosis of the lower limb during the lateral decubitus position are also avoided.[7] The abducted arms on the side of surgery in the supine position can be adducted intermittently unlike the fixed posture in the lateral decubitus position. By taking advantage of the anatomical location of the mediastinal tumour, further displacement of lung tissue on the side of the lesion can be achieved by a slight reverse Trendelenburg position which shifts the lung tissue further away from the lesion. Postoperatively because of a small mini-axillary thoracotomy wound, pain control is adequate with non-narcotics resulting in early patient mobilization as well as early discharge from the hospital as in VATS, a cosmetically sited surgical scar which is small enough to be hidden in the axilla or the upper arm is present [Figure 5].
